# Ectopic expression of human *TUBB8* leads to increased aneuploidy in mouse oocytes

**DOI:** 10.1038/s41421-023-00599-z

**Published:** 2023-10-24

**Authors:** Jie Dong, Liping Jin, Shihua Bao, Biaobang Chen, Yang Zeng, Yuxi Luo, Xingzhu Du, Qing Sang, Tianyu Wu, Lei Wang

**Affiliations:** 1 Institute of Pediatrics, Children’s Hospital of Fudan University and Institutes of Biomedical Sciences, The State Key Laboratory of Genetic Engineering, Fudan University, Shanghai, China; 2 Shanghai Key Laboratory of Maternal Fetal Medicine, Clinical and Translational Research Center, Shanghai First Maternity and Infant Hospital, School of Medicine, Tongji University, Shanghai, China; 3 Department of Reproductive Immunology, Shanghai First Maternity and Infant Hospital, School of Medicine, Tongji University, Shanghai, China; 4 NHC Key Lab of Reproduction Regulation, Shanghai Institute for Biomedical and Pharmaceutical Technologies, Fudan University, Shanghai, China

**Keywords:** Developmental biology, Meiosis

## Abstract

Aneuploidy seriously compromises female fertility and increases incidence of birth defects. Rates of aneuploidy in human eggs from even young women are significantly higher than those in other mammals. However, intrinsic genetic factors underlying this high incidence of aneuploidy in human eggs remain largely unknown. Here, we found that ectopic expression of human *TUBB8* in mouse oocytes increases rates of aneuploidy by causing kinetochore–microtubule (K–MT) attachment defects. Stretched bivalents in mouse oocytes expressing *TUBB8* are under less tension, resulting in continuous phosphorylation status of HEC1 by AURKB/C at late metaphase I that impairs the established correct K–MT attachments. This reduced tension in stretched bivalents likely correlates with decreased recruitment of KIF11 on meiotic spindles. We also found that ectopic expression of *TUBB8* without its C-terminal tail decreases aneuploidy rates by reducing erroneous K–MT attachments. Importantly, variants in the C-terminal tail of *TUBB8* were identified in patients with recurrent miscarriages. Ectopic expression of an identified *TUBB8* variant in mouse oocytes also compromises K–MT attachments and increases aneuploidy rates. In conclusion, our study provides novel understanding for physiological mechanisms of aneuploidy in human eggs as well as for pathophysiological mechanisms involved in recurrent miscarriages.

## Introduction

Human eggs frequently contain an incorrect number of chromosomes, a condition termed aneuploidy which acts as one of the major causes for female infertility/subfertility, recurrent miscarriages and birth defects^1,2^. The incidence of aneuploid eggs in women aged 20–32 years is 20%–30%^3,4^, which is higher than many other species in reproductive age, such as mouse (1%–4%)^4–6^, porcine (3%–10%)^4,7,8^, bovine (5.8%–7.1%)^9,10^, equine (~15.6%)^4,11^ and rhesus macaque (7.1%–18.4%)^12^. Although high frequency of aneuploidy has been observed in human eggs, specific genetic factors and precise mechanisms causing this high rate of aneuploidy in human eggs under physiological conditions remain to be elucidated.

Aneuploidy of human eggs is caused by chromosome segregation errors in meiosis I^13^. Studies have demonstrated that tri-directional division induced by transient tripolar spindles could cause error-prone chromosome segregation in the anaphases^14^. The absence of KIFC1 in human oocytes has been suggested to contribute to their unstable and multiple spindle poles, leading to an increase in aneuploidy^15^. Also, the transient unstable multipolar spindles cause abnormal kinetochore–microtubule (K–MT) attachments in human oocytes, which cause chromosome missegregation^1,16^. The paired kinetochores are attached by microtubules from opposite spindle poles to ensure accurate chromosome segregations^17^. Stable kinetochore microtubules (also known as k-fiber) are required for correct K–MT attachments. The microtubules are commonly composed of conserved α- and β-tubulin heterodimers^18,19^. Human β-tubulin consists of nine isotypes, including TUBB1, TUBB2A, TUBB2B, TUBB3, TUBB4A, TUBB4B, TUBB5, TUBB6, and TUBB8; and eight of them are also detected in mouse oocytes, with the exception of TUBB8^18–20^. We previously demonstrated that TUBB8 is uniquely expressed in human oocyte spindles, which consists of microtubules polymerized from heterodimers containing the preponderant β-tubulin isotype encoded by *TUBB8*^21^. In contrast, mouse oocyte spindles are mainly made up of β-tubulin isotypes such as TUBB3, TUBB4B, TUBB5 and TUBB6. In addition, we previously showed that patients with variants in *TUBB8* exhibit oocyte meiotic arrest, early embryonic development arrest or aneuploidy caused by a broad spectrum of spindle defects^21–24^. Considering that *TUBB8* is a primate-specific gene and plays a vital role in spindle microtubule polymerization during human oocyte meiosis, we hypothesized that TUBB8 may contribute to the high aneuploidy rate in human eggs.

In this study, we demonstrate that ectopic expression of primate-specific *TUBB8* induces aneuploidy escalation in mouse oocytes, and further clarified the mechanisms of aneuploidy induced by *TUBB8*. In addition, we showed that variants in the C-terminal tail of *TUBB8* are more prone for producing aneuploid eggs and associated with recurrent miscarriages. These results provide a novel understanding of the physiological and pathophysiological mechanisms of high frequency of aneuploidy in human eggs.

## Results

### *TUBB8* increases the incidence of aneuploidy in mouse eggs

In order to determine whether TUBB8 promotes aneuploidy in human eggs, we firstly microinjected varying concentrations of human *TUBB8* capped RNA (cRNA) into mouse germinal vesicle (GV) oocytes to observe its influence on oocyte maturation. Compared to vehicle controls, oocytes injected with 200 ng/μL *TUBB8* cRNA took a longer time for the first polar body (PB1) to be extruded (Supplementary Fig. S1a) and displayed smaller spindles (Supplementary Fig. S1b), which is similar with human oocytes. Oocytes injected with a low concentration (100 ng/μL) of cRNA made no significant difference compared to vehicle controls (Supplementary Fig. S1a), while oocytes injected with a higher concentration (500 ng/μL) of cRNA were primarily arrested at metaphase I (MI) due to spindle assembly defects (Supplementary Fig. S1a). Therefore, the concentration of 200 ng/μL was chosen for subsequent experiments. We next asked if these phenotypes are indeed induced by ectopic expression of *TUBB8*. Considering that TUBB5 and TUBB4A display extensive homologies to TUBB8, and TUBA1C is the most highly expressed α-tubulin in human oocytes and embryos, we chose to microinject the same concentration of human *TUBB5*, *TUBB4A*, and *TUBA1C* cRNA into mouse oocytes, respectively. These human tubulin isotypes were equally well incorporated into the MI spindles (Supplementary Fig. S1b, c). Compared to oocytes expressing other α- or β-tubulins isotypes, oocytes expressing *TUBB8* showed no difference in microtubule densities (Supplementary Fig. S1d), but displayed less microtubule mass (Supplementary Fig. S1e), shorter spindle lengths (Supplementary Fig. S1b, f) and smaller spindle volumes (Supplementary Fig. S1b, g).

**Fig. 1:**
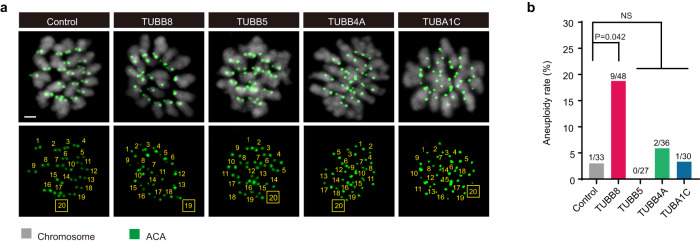
Ectopic expression of *TUBB8* increased incidence of aneuploidy in mouse oocytes. **a** Representative images of euploid and aneuploid MII oocytes expressing different human tubulin isotypes. Control indicates mouse GV oocytes injected with vehicle; TUBB8, TUBB5, TUBB4A and TUBA1C indicate mouse GV oocytes injected with the corresponding 5’FLAG-cRNA (200 ng/μL) of human tubulin isotypes, respectively. Mouse GV oocytes were maintained in 2.5 μM milrinone during microinjection and then immediately released into milrinone-free M2 medium to allow the resumption of meiosis. Chromosome spreads were treated with monastrol (100 μM) at 16 h after NEBD, and then oocytes were fixed and immunostained for centromere (ACA) and DNA (using Hoechst). Chromosome numbers are indicated in the images. Scale bar, 2 μm. **b** Aneuploidy rates of MII oocytes expressing different human tubulin isotypes. Fisher’s exact test. NS, not significant. Data were collected from at least three independent experiments.

We then evaluated the proportion of aneuploid eggs expressing *TUBB8*. The rate of aneuploidy in metaphase II (MII) oocytes expressing *TUBB8* was significantly higher than that in those expressing *TUBB5*, *TUBB4A*, or *TUBA1C* (18.75% vs. up to 5.56%) (Fig. 1a, b). These results suggest that TUBB8 could induce increased rates of aneuploidy during female meiosis.

### *TUBB8* delays meiotic progression in mouse oocytes mainly through spindle assembly checkpoint (SAC) activation

According to previous investigations, oocyte aneuploidy is highly correlated with accelerated meiotic progression, which was caused by compromised SAC function^25^, Oocytes with SAC defects lose the supervision of K–MT attachments and accelerate the progression of meiosis^26–28^. Accordingly, to figure out whether TUBB8-induced aneuploidy of mouse oocytes was associated with impaired SAC function, we initially test the progression of meiosis I.

**Fig. 2:**
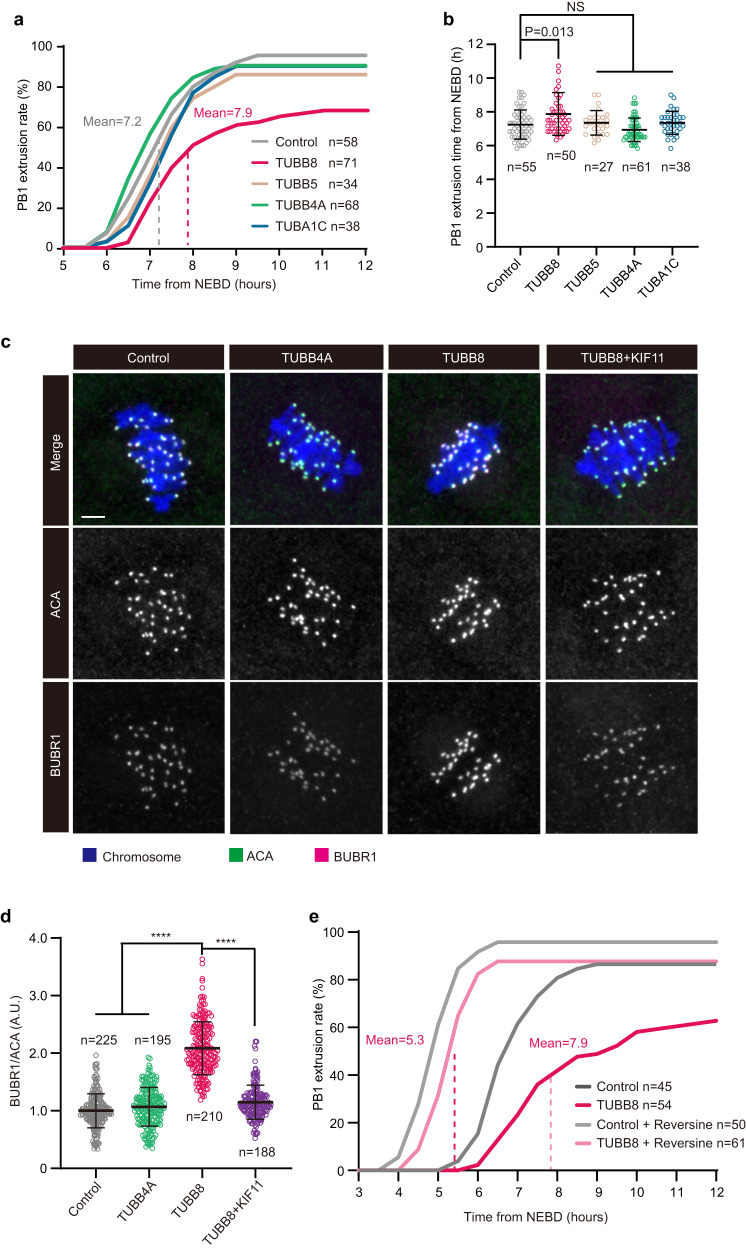
Ectopic expression of *TUBB8* delayed meiotic progression mainly by SAC activation. **a** Statistical analysis of PB1 extrusion rates in mouse oocytes expressing different human tubulin isotypes at consecutive time points after NEBD. *n*, number of oocytes. **b** Statistical analysis of PB1 extrusion time in mouse oocytes expressing different human tubulin isotypes. *n*, number of oocytes. One-way ANOVA with multiple comparisons test. NS, not significant. **c** Representative images of SAC activity as indicated by localization of BUBR1 at late MI in mouse oocytes expressing different human tubulin isotypes. BUBR1 signal was shown in a separate channel. Mouse GV oocytes were injected with vehicle, 5’FLAG*-TUBB4A*-cRNA (200 ng/μL), 5’FLAG-*TUBB8*-cRNA (200 ng/μL), or a combination of 5’FLAG-*KIF11* (1000 ng/μL) and 5’FLAG-*TUBB8*-cRNA (400 ng/μL), maintained for 1 h in 2.5 μM milrinone, and then washed and transferred into milrinone-free M2 medium to allow the resumption of meiosis. At 5 h after NEBD, oocytes were fixed and immunostained for BUBR1, ACA, and DNA (using Hoechst), Scale bar, 5 μm. **d** Relative intensities of BUBR1 normalized to signal intensities of ACA. *n*, number of kinetochores. One-way ANOVA with multiple comparisons test. ****P < 0.0001. **e** Statistical analysis of PB1 extrusion rates at consecutive time points after NEBD for controls and mouse oocytes expressing *TUBB8* with or without reversine (100 nM) treatment. *n*, number of oocytes. Data in **a**, **b**, **d**, and **e** were collected from at least three independent experiments.

Compared to vehicle controls and oocytes expressing *TUBB5*, *TUBB4A*, or *TUBA1C*, the mean time of PB1 extrusion after nuclear envelope breakdown (NEBD) in mouse oocytes expressing *TUBB8* was significantly delayed (~7.9 h vs. 7.2 h; Fig. 2a, b), and rate of PB1 extrusion was also slightly decreased in mouse oocytes expressing *TUBB8* (Fig. 2a). These results suggest that function of SAC remains intact, but may not be properly inactivated at late MI, resulting in a delay in meiotic progression.

To test if the delayed meiotic progression was induced by SAC activation, we examined the localization of BUBR1, a key component of SAC^26,29^, by immunofluorescence staining. BUBR1 is recruited to kinetochores early in prometaphase after NEBD, and most of BUBR1 signal decreases or disappears at metaphase when all kinetochores are attached by spindle microtubules properly^30^. As indicated in Fig. 2c, d, BUBR1 is recruited to kinetochores at late MI and signal intensities of BUBR1 showed no changes in oocytes expressing *TUBB8*, indicating that SAC was not properly inactivated as in control oocytes, but still maintained activated.

To further confirm whether the meiotic delay is mediated by SAC, mouse oocytes expressing *TUBB8* were further treated with reversine, a small molecule inhibitor of the mitotic kinase MPS1 that initiates SAC response^31^, that can prevent SAC-mediated meiotic delay of mouse oocytes^32^. As shown in Fig. 2e, the mean time of PB1 extrusion in mouse oocytes expressing *TUBB8* was accelerated with reversine treatment (5.3 h vs. 7.9 h). These results suggest that TUBB8 delayed meiotic progression in mouse oocytes mainly by SAC activation.

### *TUBB8* induces erroneous K–MT attachments through reducing the tension of stretched bivalents in mouse oocytes

**Fig. 3:**
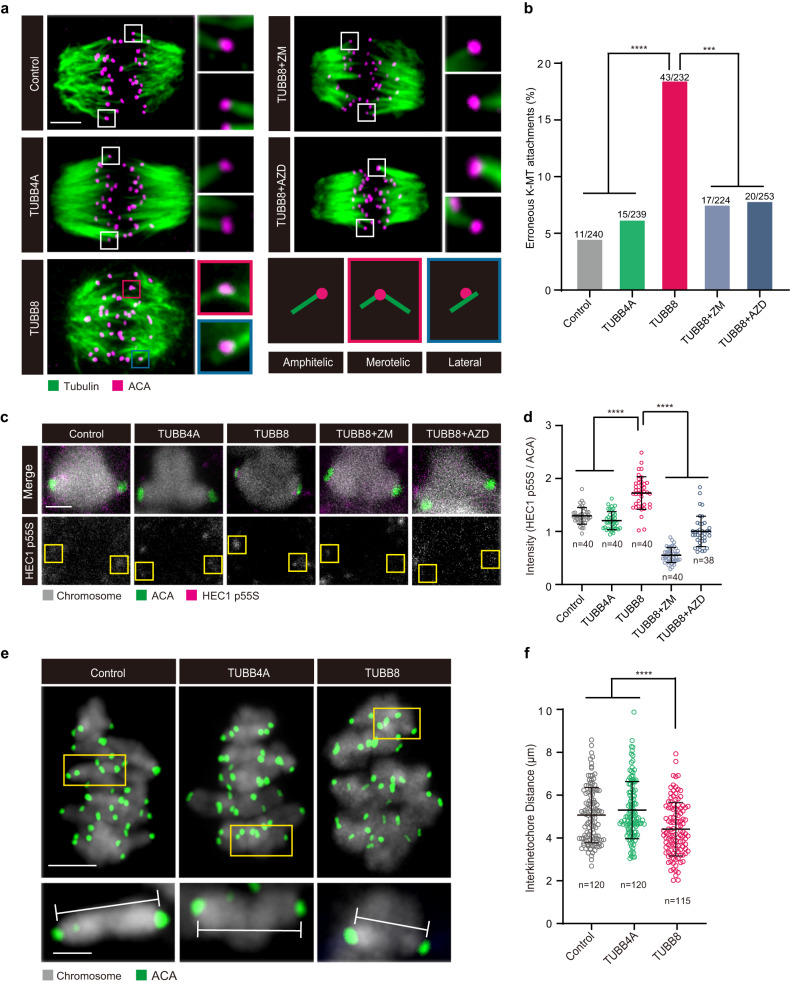
Ectopic expression of *TUBB8* induced erroneous K–MT attachments by reducing tension of stretched bivalents in mouse oocytes. **a** Representative images of K–MT attachments, including amphitelic attachments, merotelic attachments, and lateral interactions in mouse oocytes expressing different human tubulin isotypes. Mouse GV oocytes were injected with vehicle, 5’FLAG-*TUBB4A*-cRNA (200 ng/μL), or 5’FLAG-*TUBB8*-cRNA (200 ng/μL), maintained for 1 h in 2.5 μM milrinone, and then washed and transferred into milrinone-free M2 medium to allow the resumption of meiosis. At 5 h after NEBD, oocytes were placed on ice for 10 min to induce depolymerization of unstable microtubules and then immediately fixed and immunostained for tubulin, ACA, and DNA (using Hoechst). Where indicated, oocytes injected with *TUBB8* cRNA at 5 h after NEBD were treated either with ZM447439 (10 μM) or AZD1152 (500 nM) for 15 min or 30 min prior to the cold treatment. Scale bar, 5 μm. **b** Percentages of erroneous K–MT attachments in mouse oocytes expressing different human tubulin isotypes. Fisher’s exact test. ***P < 0.001, ****P < 0.0001. **c** Representative images show HEC1 pS55 (phosphorylation of HEC1 on Ser55) at late MI stage in mouse oocytes expressing different human tubulin isotypes. Oocytes injected with *TUBB8* cRNA were treated either with ZM447439 (10 μM) or AZD1152 (500 nM) for 15 min or 30 min to inhibit AURKB/C and then fixed and immunostained for HEC1 pS55, ACA, and DNA (using Hoechst). Scale bar, 2 μm. **d** Relative intensities of HEC1 pS55 normalized to that of ACA. *n*, number of kinetochores. One-way ANOVA with multiple comparisons test. ****P < 0.0001. **e** Representative full overlays of bivalents and kinetochores in the same oocytes expressing different human tubulin isotypes. Magnifications of stretched chromosomes in the different groups were shown in the yellow insets. Interkinetochore distances were determined as indicated in the magnifications. Scale bar, 5 μm (top) and 2 μm (bottom). **f** Distribution of interkinetochore distances. *n*, number of bivalents. One-way ANOVA with multiple comparisons test. ****P < 0.0001. Data in **b**, **d**, and **f** were collected from at least three independent experiments.

SAC activation at late MI mostly results from the incorrect K–MT attachments^17^. To understand why SAC is not inactivated until late MI, we examined K–MT attachments in mouse MI oocytes with *TUBB8* expression. We imaged mouse oocytes with cold treatment and categorized the K–MT attachments as amphitelic attachments, merotelic attachments, and lateral interactions (Fig. 3a). The percentage of erroneous K–MT attachments including merotelic and lateral interactions in mouse oocytes expressing *TUBB8* increased to 18.5%, while the percentage was 4.6% and 6.3% in vehicle controls and in oocytes expressing *TUBB4A*, respectively (Fig. 3b). These results suggest that ectopic expression of *TUBB8* increased rates of K–MT attachment errors in mouse oocytes.

Although TUBB8-induced high incidence of incorrect K–MT attachments was observed until late MI in mouse oocytes, the mechanism is still unknown. In mouse oocytes, aurora kinase C (AURKC), as the predominant CPC (chromosomal passenger complex) kinase, is the key factor to correct erroneous K–MT attachments instead of aurora kinase B (AURKB); while AURKB can compensate for the loss of AURKC to perform K–MT attachment error correction^33–35^. AURKB/C detaches erroneously attached microtubule fibers by phosphorylating HEC1, a key protein of kinetochore-associated NDC80 complex, during prometaphase of meiosis I^36,37^.

However, AURKB/C at late MI could also phosphorylate its substrate HEC1 because kinetochores are not stretched away from aurora kinases on the centromeres^35^. Therefore, AURKB/C destabilizes correct K–MT attachments and allows a considerable number of incorrect attachments^34,37^. Therefore, we supposed that continuous phosphorylation status of HEC1 by AURKB/C at late MI may be responsible for the TUBB8-induced increase in K–MT attachment errors.

Therefore, we evaluated levels of AURKB/C-dependent phosphorylation of HEC1 on Ser55 in oocytes expressing *TUBB8* or *TUBB4A* and in vehicle controls. A significant increase in HEC1 phosphorylation in mouse oocytes expressing *TUBB8* was observed (Fig. 3c, d), suggesting that AURKB/C still continuously phosphorylates HEC1 even at late MI.

To confirm that the increased level of HEC1 phosphorylation in oocytes expressing *TUBB8* indeed resulted from AURKB/C at late MI, we used the drug ZM447439 and AZD1152 to inhibit AURKB/C, respectively. The short-term treatment by ZM447439 at a concentration of 10 μM and AZD1152 at a concentration of 500 nM has been reported to cause efficient inhibition of AURKB/C in mouse oocytes^37,38^. We also ruled out the possibility that AURKA was concomitantly inhibited in mouse oocytes upon the same treatment (Supplementary Fig. S2a, b). Our results showed that phosphorylation of HEC1 on Ser55 by AURKB/C in oocytes expressing *TUBB8* was significantly reduced after inhibiting AURKB/C (Fig. 3c, d), and percentages of erroneous attachments dropped to 7.6% and 7.9%, respectively, which was comparable to vehicle controls and to *TUBB4A*-expressing oocytes (Fig. 3a, b).

In mitosis, as sister kinetochores bind to microtubules from opposing spindle poles, tension is generated, actively stretching the kinetochore away from the centromere. Chromosomes that are bi-oriented contain the most tension, physically limiting AURKB from phosphorylating kinetochore-bound substrates. Reduced tension allows AURKB on the centromeres to phosphorylate HEC1 on the kinetochores, and ultimately destabilizing the attachments^35^. Additionally, a recent study suggested that less stretched bivalents allow the continuous phosphorylation of HEC1 by AURKB/C, resulting in transient microtubule detachment and formation of erroneous K–MT attachments such as lateral interactions^38^. We therefore measured the distance between pairs of sister kinetochores (the interkinetochore distance) from each bivalent in mouse oocytes expressing *TUBB8* or *TUBB4A*. As indicated in Fig. 3e, f, the interkinetochore distance was shorter in oocytes expressing *TUBB8*, indicating that reduced stretching of bivalents was caused by *TUBB8*. These results demonstrated that ectopic expression of *TUBB8* could induce continuous phosphorylation status of HEC1 by AURKB/C through reducing the tension of stretched bivalents, further resulting in erroneous K–MT attachments.

### The reduced tension in stretched bivalents in mouse oocytes expressing *TUBB8* may be correlated with decreased recruitment of KIF11 on MI spindles

**Fig. 4:**
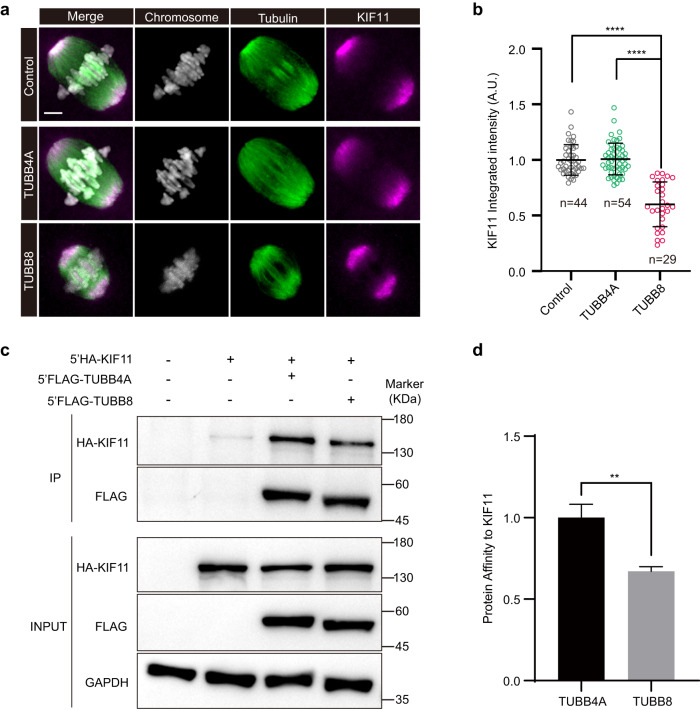
Less stretched bivalents caused by ectopic expression of *TUBB8* may be correlated with reduced recruitment of KIF11 on MI spindles in mouse oocytes. **a** Representative images of KIF11 localization on spindles in MI mouse oocytes expressing different human tubulin isotypes. Mouse GV oocytes were injected with vehicle, 5’FLAG-*TUBB4A* (200 ng/μL), or 5’FLAG-*TUBB8*-cRNA (200 ng/μL), maintained for 1 h in 2.5 μM milrinone, and then washed and transferred into milrinone-free M2 medium to allow the resumption of meiosis. At 5 h after NEBD, oocytes were fixed and immunostained for tubulin, KIF11, and DNA (using Hoechst). Scale bar, 5 μm. **b** Relative levels of KIF11 integrated intensities in areas of spindles of mouse oocytes expressing different human tubulin isotypes. *n*, number of oocytes. One-way ANOVA with multiple comparisons test. ****P < 0.0001. **c** Protein affinity of TUBB4A and TUBB8 for KIF11. HEK293T cells were co-transfected with 5’HA-*KIF11* plasmid together with 5’FLAG-*TUBB4A* or 5’FLAG-*TUBB8* plasmid and subjected to co-immunoprecipitation using anti-FLAG antibody. The interaction between 5’FLAG-*TUBB4A* or 5’FLAG-*TUBB8* with 5’HA-*KIF11* was shown by the presence of 5’HA-*KIF11* in the immunoprecipitate eluate pulled down by anti-FLAG antibody. **d** Statistical analysis of protein affinity for KIF11. Student’s *t*-test. **P < 0.01. Data in **b** and **d** were collected from at least three independent experiments.

KIF11 (kinesin family member 11) is a molecular motor that crosslinks anti-parallel microtubules and maintains bipolar spindle structure in MI mouse oocytes^39^. A recent study showed that the tension of stretched bivalents in mouse oocytes was significantly reduced upon inactivation of KIF11 by a low concentration of s-trityl-l-cysteine (STLC), a small molecule inhibitor of KIF11^38^. We thus measured levels of KIF11 on MI spindles in oocytes expressing *TUBB8*. KIF11 was located in the spindle poles of MI oocytes (Fig. 4a), and the recruitment of KIF11 on MI spindles, as indicated by integrated intensities, was significantly reduced in oocytes expressing *TUBB8* (Fig. 4b). Additionally, co-immunoprecipitation assay using HEK293T cells demonstrated that TUBB8 displayed less affinity for KIF11 compared with TUBB4A (Fig. 4c, d).

To further confirm whether KIF11 contributes to the less stretched bivalents in oocytes expressing *TUBB8*, human *KIF11* cRNA (500 ng/μL) was injected into GV oocytes expressing *TUBB8*. Aberrant interkinetochore distance and spindle length were rescued (Supplementary Fig. S3a, d, e), and signal intensities of SAC protein BUBR1 was also reduced to levels similar to vehicle controls (Fig. 2c, d). In addition, the interkinetochore distance and spindle length were shorter in mouse oocytes treated with STLC (Supplementary Fig. S3a–c). Compared to oocytes expressing *TUBB8*, interkinetochore distance showed no difference and spindle length became shorter in oocytes expressing *TUBB8* after STLC treatment (Supplementary Fig. S3a, d, e). These results suggest that the less stretched bivalents in oocytes with ectopic expression of *TUBB8* may be correlated with the reduced recruitment of KIF11 on MI spindles. Furthermore, expressing human *KIF11* in mouse oocytes expressing *TUBB8* partly rescued the reduced tension of stretched bivalents and the SAC activation caused by ectopic expression of *TUBB8*.

### Variants in *TUBB8* C-terminal tail are significantly associated with high frequency of aneuploidy

**Fig. 5:**
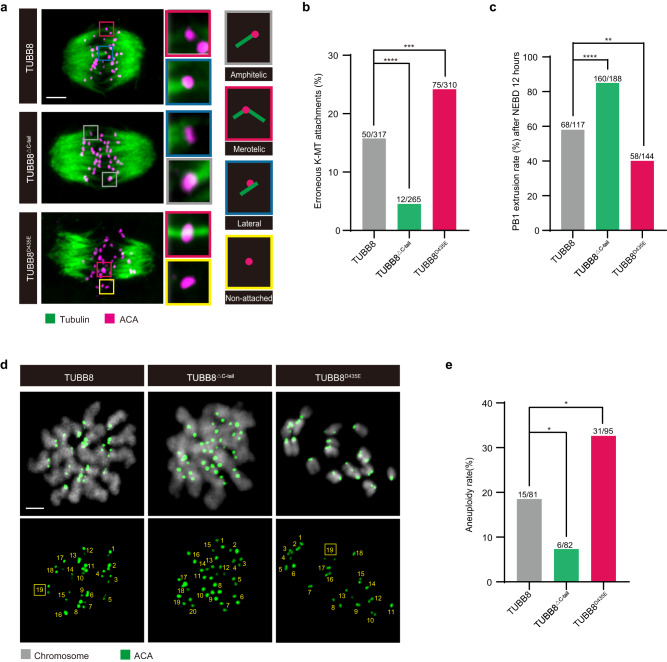
Variants in *TUBB8* C-terminal tail were associated with increased frequencies of aneuploidy in mouse oocytes. **a** Representative images of K–MT attachments, including amphitelic attachments, merotelic attachments, lateral interactions, and non-attached kinetochores in mouse oocytes expressing full-length *TUBB8*, *TUBB8* without C-terminal tail and the D435E variant. Mouse GV oocytes were injected with 5’FLAG-*TUBB8* cRNA, 5’FLAG-*TUBB8*^△C-tail^ cRNA, or 5’FLAG- *TUBB8*^D435E^ cRNA (200 ng/μL), maintained for 1 h in 2.5 μM milrinone, and then washed and transferred into milrinone-free M2 medium to allow the resumption of meiosis. At 5 h after NEBD, oocytes were placed on ice for 10 min to induce depolymerization of unstable microtubules and then immediately fixed and immunostained for tubulin, ACA, and DNA (using Hoechst). Scale bar, 5 μm. **b** Percentages of erroneous K–MT attachments in mouse oocytes expressing full-length *TUBB8*, *TUBB8* without C-terminal tail and the D435E variant. **c** PB1 extrusion rates in mouse oocytes expressing full-length *TUBB8*, *TUBB8* without C-terminal tail and the D435E variant at 12 h after NEBD. Mouse GV oocytes were injected with 5’FLAG-*TUBB8* cRNA, 5’FLAG-*TUBB8*^△C-tail^ cRNA, or 5’FLAG-*TUBB8*^D435E^ cRNA (200 ng/μL), maintained in 2.5 μM milrinone during the injection, and then washed and transferred into milrinone-free M2 medium to allow the resumption of meiosis. At 12 h after NEBD, PB1 extrusion rates of different groups were analyzed. **d** Representative images of euploid and aneuploid MII oocytes expressing full-length *TUBB8*, *TUBB8* without C-terminal tail and the D435E variant. Chromosome spreads were treated with monastrol (100 μM) at 16 h after NEBD in different groups, and then oocytes were fixed and immunostained for ACA and DNA (using Hoechst). Chromosome numbers are indicated in the images. Scale bar, 2 μm. **e** Aneuploidy rates of MII oocytes expressing full-length *TUBB8*, *TUBB8* without C-terminal tail and the D435E variant. Fisher’s exact test (**b**, **c**, and **e**). *P < 0.05, **P < 0.01, ***P < 0.001, ****P < 0.0001. Data in **b**, **c**, and **e** were collected from at least three independent experiments.

Amino acids 1–430 of the nine human β-tubulin isotypes (TUBB1, TUBB2A, TUBB2B, TUBB3, TUBB4A, TUBB4B, TUBB5, TUBB6, and TUBB8) are highly conserved, but their amino acids in C-terminal tails show great divergence^18,19^. To further investigate the causal relationship between the C-terminal tail of *TUBB8* and aneuploidy in eggs, cRNA of *TUBB8* without the C-terminal tail (*TUBB8*^△C-tail^) was injected into mouse GV oocytes. *TUBB8* without the C-terminal tail was incorporated into spindles equally well as full-length *TUBB8* (Supplementary Fig. S4a–c). Compared to ectopic expression of full-length *TUBB8*, the rate of incorrect K–MT attachments in mouse oocytes expressing *TUBB8*^△C-tail^ was reduced to 4.5% (Fig. 5a, b), resulting in the frequency of aneuploidy decreasing to 7.3% (Fig. 5d, e) and the rate of PB1 extrusion increasing to 85.1% (Fig. 5c). This further indicated that the C-terminal tail played a role in the unique function of TUBB8.

**Fig. 6:**
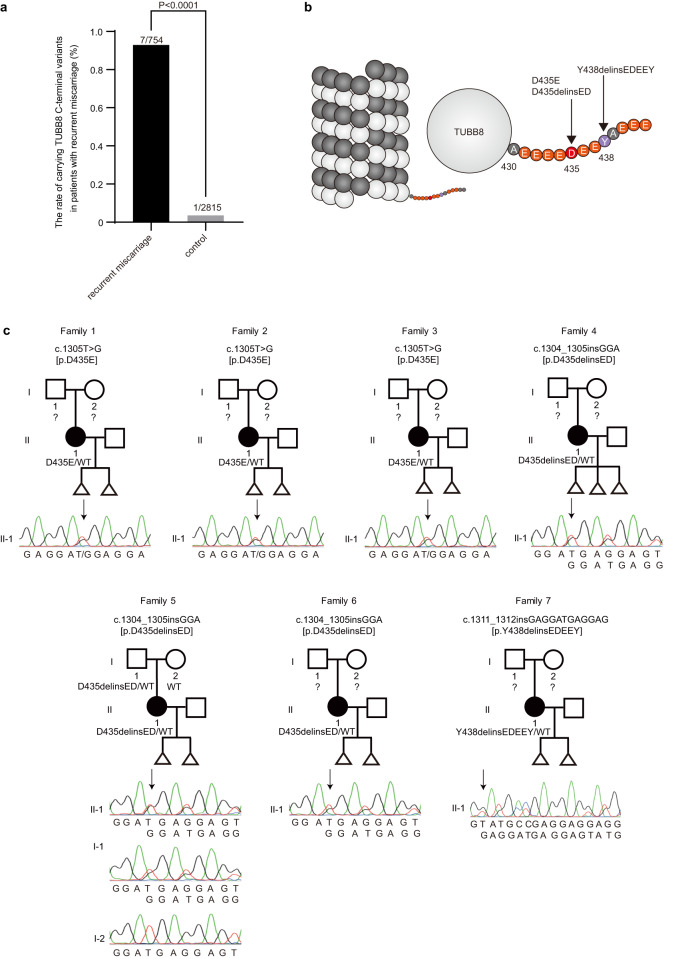
Variants in *TUBB8* C-terminal tail were identified in patients with recurrent miscarriages. **a** Rates of carrying *TUBB8* C-terminal variants in patients with recurrent miscarriages and in control subjects. Fisher’s exact test. **b** Schematic diagram of the *TUBB8* C-terminal tail. Arrowheads indicate the rare variants at amino acids of 435 and 438 in patients with recurrent miscarriages. **c** All variants were confirmed by Sanger sequencing. Squares indicate males, and circles indicate females. Black circles indicate affected individuals, and triangles indicate aborted fetuses. Question marks refer to unavailable DNA samples.

In clinics, it has been shown that ~50% of spontaneously aborted fetuses are aneuploid^40,41^. We therefore performed genetic screening of variants in *TUBB8* in 754 patients characterized by recurrent miscarriages. As indicated in Fig. 6a, compared to controls, there was a significant enrichment of rare variants in the *TUBB8* C-terminal tail in recurrent miscarriage patients (7/754 patients vs. 1/2815 controls, *P* < 0.0001). Among the seven patients with *TUBB8* rare variants, a D435E variant was found in three patients, other three patients had recurrent D435delinsED variant, and one patient had the Y438delinsEDEEY variant (Fig. 6b). All variants were confirmed by Sanger sequencing (Fig. 6c). Then, cRNA of *TUBB8* with the D435E variant (*TUBB8*^D435E^) was injected into mouse GV oocytes to evaluate the resulting phenotypes. The *TUBB8* with the D435E variant was incorporated into spindles equally well as full-length *TUBB8* and as *TUBB8* without the C-terminal tail. (Supplementary Fig. S4a–c). Expressing *TUBB8*^D435E^ in mouse oocytes caused an increased incidence of errors in K–MT attachments (24.2%), including a few unattached kinetochores (Fig. 5a, b), a higher rate of aneuploid eggs (32.6%) (Fig. 5d, e), and a reduced rate of PB1 extrusion (40.3%) (Fig. 5c). These results suggested that rare variants in the C-terminal tail of *TUBB8* may be associated with a higher proportion of aneuploid eggs in recurrent miscarriage patients.

## Discussion

In this study, we figured out the essential role of the primate-specific gene *TUBB8* in the high rate of aneuploidy in human eggs. We found that ectopic expression of *TUBB8* could delay meiotic progression and increase the incidence of aneuploidy in mouse eggs. In addition, ectopic expression of *TUBB8* in mouse oocytes led to reduced tension on the stretched bivalents. This induced continuous phosphorylation status of HEC1 by AURKB/C at late MI, resulting in erroneous K–MT attachments and ultimately increasing aneuploidy rates in mouse eggs and delaying meiotic progression. And the reduced tension in stretched bivalents in mouse oocytes expressing *TUBB8* may be correlated with reduced recruitment of KIF11 on MI spindles. Importantly, the C-terminal tail of *TUBB8* made a contribution to increased aneuploidy in eggs.

Compared with other species, a dramatically higher level of aneuploidy is a unique characteristic of eggs from women of reproductive age. We recently discovered a unique microtubule structure in human oocytes, which we named human oocyte microtubule organizing center (huoMTOC). The huoMTOC initiates microtubule nucleation and organizes the meiotic spindle apparatus in human oocytes^42^. The spindles undergo prolonged periods of multipolarity during female meiosis I, resulting in kinetochores frequently becoming attached to more than one pole and ultimately leading to incorrect merotelic attachments^1,16^. Besides, a recent study showed that deficiency in KIFC1 in human oocytes causes spindle instability and increases chromosome segregation errors^15^. However, the genetic factors responsible for K–MT attachment defects that causes chromosome segregation errors and aneuploidy of human eggs are still largely unknown. Correct K–MT attachments are maintained by stable kinetochore microtubules^17^, which are composed of conserved α- and β-tubulin heterodimers^19,20^. Previously, we demonstrated that the primate-specific gene *TUBB8*, as a β-tubulin expressing in a highly oocyte-specific manner^21^, plays a vital role in human spindle assembly and is involved in normal oocyte maturation, fertilization, and early embryonic development^21–24^. Together, these results imply that *TUBB8* may contribute to female aneuploid eggs by affecting meiotic spindle assembly.

We found that the reduced tension in stretched bivalents caused by human *TUBB8* may be correlated with decreased recruitment of KIF11 on MI spindles, and injecting human *KIF11* cRNA into mouse oocytes expressing *TUBB8* could partly rescue the phenotypes including shortened spindle length, reduced tension on stretched bivalents, and SAC activation. However, we can’t exclude the possibility that the decreased recruitment of KIF11 on MI spindles in mouse oocytes may be a consequence of reduced microtubule mass. Consequently, decreased level of KIF11 is only one of the important factors responsible for less stretched bivalents, and it is likely that other signaling pathways remain to be identified and investigated. Additionally, ectopic expression of human *KIF11* in mouse oocytes also had effects such as longer spindle length (Supplementary Fig. S3a–c), which implies that there are unknown effects of KIF11 on morphology of the spindle.

It is known that functions of different tubulin isotypes are largely affected by their C-terminal tails^19^. In this study, ectopic expression of *TUBB8* without its C-terminal tail could rescue the decreased rates of PB1 extrusion (Fig. 5c) and the increased rates of aneuploid eggs (Fig. 5d, e) by reducing errors in K–MT attachments (Fig. 5a, b). These results suggest that the C-terminal tail of *TUBB8* (amino acids 431–444) plays a role in TUBB8-mediated regulation of spindle assembly and aneuploidy escalation. However, we did not determine the exact molecular mechanisms for C-terminal tail of *TUBB8*. A number of studies have shown that C-terminal tails of tubulins possess complex and different post-translational modifications (PTMs). These PTMs, including phosphorylation, detyrosination-tyrosination, (poly) glutamylation and (poly) glycylation can alter tubulin molecules and influence MT dynamics, motor protein interactions and microtubule associated protein binding^18–20,43^. In addition, defects in tubulin PTMs can cause human diseases. For instance, deregulation of detyrosination-tyrosination cycle generated in the C-terminal tail has been shown to influence tumorigenesis and impede proper chromosome segregation during mitosis^18^. We analyzed the sequence of C-terminal tail and speculated that 438Y might be a major modification sites for phosphorylation and detyrosination-tyrosination modifications. Whether there are unique post-translational modifications in the *TUBB8* C-terminal tail as well as their corresponding variants is an open question and is worthy of further investigations.

We also have constructed an in vivo mouse model with oocyte-specific ectopic expression of 5’FLAG-Human-*TUBB8* using a *Rosa26*-targeted *ZP3* promoter-driven knockin system (Supplementary Fig. S5a). Unexpectedly, the heterozygous female mice expressing human *TUBB8* were completely infertile (Supplementary Fig. S5b), and their oocytes were arrested at MI (Supplementary Fig. S5c) because the spindle was unable to assemble (Supplementary Fig. S5d). We supposed that these serious phenotypes were caused by a high dose of *TUBB8* expression, which was consistent with the phenotypes in oocytes injected with *TUBB8* cRNA at a concentration of 500 ng/μL in vitro. This interesting phenomenon is worth of being investigated in the future.

Humanized mouse model has been a vital tool to study unique functions of primate-specific genes in human biology and pathology^44,45^. In our study, the primate-specific gene *TUBB8* was ectopically expressed in mouse oocytes, constructing an in vitro model for studying functions of *TUBB8*. However, this model has some limitations. Since *TUBB8* is not encoded in mouse genome, functions of *TUBB8* may be compensated by other still unidentified isotypes/proteins in mouse oocytes, making the interaction between *TUBB8* and the unidentified factor(s) more complicated. Besides, we observed different effects upon different concentrations of *TUBB8* cRNA injection (Fig. S1a). This might be caused by the relative level of ectopically expressed *TUBB8* to endogenous mouse β-tubulin. There is also a possibility that high TUBB8 expression will impair or inhibit other α- or β-tubulin isotype assembly by gain of function or dominant negative effects.

In conclusion, our study uncovers novel physiological and pathophysiological roles for the primate-specific gene *TUBB8* in high rates of aneuploidy in human eggs caused by K–MT attachment defects by using the mouse oocyte model. This not only provides novel mechanistic insights into the unique features of high aneuploidy in human eggs, but also helps us understand diseases such as recurrent miscarriages and birth defects caused by aneuploid eggs.

## Materials and methods

### Expression plasmid construction and cRNA transcription

Full-length sequences encoding human *TUBB8* (NM_177987.3), *TUBB4A* (NM_006087.4), *TUBB5* (NM_178014.4), *TUBA1C* (NM_032704.5), and *KIF11* (NM_004523.4), and sequence of *TUBB8* without its C-terminal tail (amino acids 1–430, *TUBB8*^△C-tail^) were amplified and cloned into the PCR3.1 vector with a FLAG tag at the N-terminus to express the corresponding FLAG protein. Site-directed mutagenesis of *TUBB8* (*TUBB8*^D435E^) and the insertion of an HA tag into *KIF11* were performed using a KOD-Plus-Mutagenesis Kit (SMK-101, TOYOBO).

All expression constructs were linearized by digestion with the Not I restriction enzyme (R3189L, New England BioLabs) at 37 °C for 3 h. After purification, 1 μg of linearized DNA was used as a template to transcribe the corresponding cRNA using a HiScribe™ T7 ARCA mRNA Kit (with Tailing) (E2060S, New England BioLabs). The target cRNA was then purified using a RNeasy MinElute Cleanup Kit (74204, Qiagen).

### Mouse oocyte collection and microinjection

Female ICR mice (6–8 weeks old) were purchased from Beijing Vital River Laboratory Animal Technology Co. GV oocytes were isolated from the ovaries of female ICR mice 44–52 h following injection with 10 IU pregnant mare serum gonadotropin (ShuSheng). Fully grown GV oocytes were collected and cultured in M2 medium (M7167, Sigma-Aldrich) with milrinone (2.5 μM) to maintain arrest at prophase. Microinjection was performed using a Leica Hoffman microscope (DMi8, Leica) equipped with a TransferMan 4r micromanipulator, InjectMan 4, and FemtoJet 4i (Eppendorf). About 0.1%–0.3% volumes of cRNA solution were microinjected into the cytoplasm of each mouse GV oocyte. Different cRNA concentrations were as follows: 5’FLAG-*TUBB8* (200 ng/μL), 5’FLAG-*TUBB4A* (200 ng/μL), 5’FLAG-*TUBB5* (200 ng/μL), 5’FLAG-*TUBA1C* (200 ng/μL), 5’FLAG-*TUBB8*^△C-tail^ (200 ng/μL), 5’FLAG-*TUBB8*^D435E^ (200 ng/μL), and 5’FLAG-*KIF11* (500 ng/μL). Water was microinjected as a vehicle control. The injected GV oocytes were released into fresh M2 medium and further cultured on a 37 °C heating block in vitro. Oocytes were collected for subsequent analyses at indicated time points following culture.

### Immunofluorescence and confocal microscopy

Oocytes were fixed for 30 min in PBS containing 2% formaldehyde and 0.05% Triton X-100 and then permeabilized in 0.5% Triton X-100 for 20 min at room temperature. Oocytes were incubated at 4 °C overnight in a blocking buffer with 3% bovine serum albumin (B2064, Sigma-Aldrich) in PBS supplemented with 0.1% Tween-20 and 0.01% Triton X-100. Oocytes were incubated at 37 °C with following primary antibodies: anti-β-tubulin antibody (1:200 dilution, ab11309, Abcam), anti-FLAG antibody (1:50 dilution, ab245893, Abcam), anti-centromere antibody (ACA) (1:500 dilution; HCT-0100, ImmunoVision), anti-HEC1 phospho Ser55 antibody (1:200 dilution, GTX70017, GeneTex), anti-BUBR1 antibody (1:100 dilution, 612502, BD Biosciences), and anti-KIF11 antibody (1:200 dilution, HPA010568, Sigma-Aldrich). After washing, oocytes were incubated at 37 °C for 1 h with appropriate secondary antibodies as follows: goat anti-human Alexa Fluor 488 (1:500 dilution, 52526, Sigma-Aldrich), goat anti-rabbit Cy3 (1:500 dilution, AS007, ABclonal), and goat anti-rabbit Alexa Fluor 647 (1:500 dilution, AS060, ABclonal). DNA was counterstained with Hoechst (5 μg/mL, 1:1000 dilution, HY-15559A, MCE) for 10 min at room temperature before imaging. Finally, the oocytes were mounted on a 35-mm glass-bottom dish (801001, NEST) and images were captured using a confocal laser-scanning microscope (Leica SP8 or ZEISS LSM 880).

### Image manipulation and intensity measurements

Image manipulation and measurements were performed using Fiji-ImageJ software (National Institutes of Health, USA). The integrated fluorescence intensities on kinetochores, including ACA, BUBR1, and HEC1 (phosphor Ser55), were measured by drawing the same area using Fiji-ImageJ. The intensities of BUBR1 and HEC1 (phosphor Ser55) were normalized against the ACA intensities. Integrated intensities of KIF11 in Fig. 4 were measured in the areas of the spindles in every oocyte. Signal intensities of AURKA pT288 in Supplementary Fig. S2 were measured relative to tubulin. 3D image visualization and spindle volume calculations in Supplementary Fig. S1 were performed using Imaris 9.6 (Bitplane, Switzerland).

### Cold treatment for microtubule depolymerization

Oocytes were placed on ice for 10 min to depolymerize non-K–MTs. The oocytes were immediately fixed and processed for immunofluorescence as described above.

### Inhibitor treatments

Monastrol (100 μM, HY101071A, MCE) was added to M2 medium of MII oocytes for 1.5 h for chromosome spreading. Reversine (100 nM, HY-14711, MCE) was added to oocytes starting from NEBD to inhibit SAC activity. ZM447439 (10 μM, HY-10128, MCE) or AZD1152 (500 nM, SML0268, Sigma-Aldrich) was used to treat oocytes for 15 min or 30 min at 5 h after NEBD to inhibit AURKB/C. S-Trityl-L-cystein (STLC) (0.75 μM, 164739, Sigma-Aldrich) was used to treat oocytes at 5 h after NEBD to inhibit KIF11.

### Cell culture, transfection, and co-immunoprecipitation

HEK293T cells were cultured in DMEM (MA0212, Meilunbio) supplemented with 10% fetal bovine serum (10199-141, Gibco) and 1% penicillin-streptomycin (15140-122, Gibco) in an atmosphere of 5% CO_2_ at 37 °C. Expression plasmids containing 5’HA-*KIF11* and plasmids containing 5’FLAG-*TUBB8* or 5’FLAG-*TUBB4A* were co-transfected into HEK293T cells using the PolyJet In Vitro DNA Transfection Reagent (SL100688, SignaGen). Proteins were extracted in NP-40 lysis buffer (0.5% NP-40, 50 mM Tris, 150 mM NaCl, pH 7.5) with 1% protease inhibitor cocktail (B14002, Bimake). Total lysate was incubated with FLAG-IgG Agrose beads (B23102, Bimake) for 3 h on a rotating wheel at 4 °C. The beads were washed with NP-40 lysis buffer for 4–5 times and then with 2× SDS loading buffer for western blotting assay.

Proteins were denatured by heating to 100 °C, separated by SDS-polyacrylamide gel electrophoresis (GSH2001, EZBiolab), and transferred to a nitrocellulose filter membrane (Pall Corporation). The membrane was blocked in 5% non-fat milk diluted in PBST buffer for 1 h, then incubated at 4 °C overnight with antibodies against FLAG (GNI4110-FG, GNI; F7425, Sigma-Aldrich), HA (3724, Cell Signaling Technology), and GAPDH (5174, Cell Signaling Technology). The membranes were washed with PBST three times and incubated for 1 h at room temperature with corresponding secondary antibodies: goat anti-mouse (1:5000 dilution, B30008, Abmart), goat anti-rabbit (1:5000 dilution, B30009, Abmart), and goat anti-rabbit, light chain specific (1:3000 dilution, 211-002-171, Jackson ImmunoResearch). The membranes were washed again three times with PBST, and the blots were imaged on an imaging workstation (Tanon 5200 s) after detection with the ECL western blotting substrate. The integrated densities of the blots were measured by Fiji-ImageJ.

### Clinical samples

The 754 patients with recurrent miscarriages in this study were recruited from the Shanghai First Maternity and Infant Hospital. The aborted fetuses in about 50% of the patients were aneuploid. The 2815 individuals in the control group conceived naturally or through IVF/ICSI cycles.

### Genetic analysis

Genomic DNA samples were extracted from peripheral blood using a MagBeads Blood DNA Extraction Midi Kit (Enriching Biotechnology, Suzhou, China). Agilent SureSelect Whole-Exome capture and Illumina sequencing technology were used to sequence the exomes of patients and control subjects. Variant analysis and annotation were performed with the Genome Analysis Toolkit and ANNOVAR. In this study, the variants in the *TUBB8* C-terminal tail were filtered according to the following criteria: (1) the frequency of variants should be less than 0.1% in the gnomAD database (http://gnomad-old.broadinstitute.org); (2) variants should be significantly enriched in patients compared with control samples; and (3) if the DNA of parents is available, the variants should satisfy Mendelian inheritance. The variants in patients were confirmed by Sanger sequencing, and the primers are listed in Supplementary Table S1.

### Statistical analysis

All tests were performed using GraphPad Prism 8 (GraphPad Software). Fisher’s exact test, one-way ANOVA with multiple comparisons or with Student’s *t*-test were performed where indicated.

### Supplementary information

TUBB8-Supplemental Materials

## Data Availability

The data and materials underlying this article will be shared upon reasonable request to the corresponding authors.
