# Supplementary material for: Ectopic expression of human TUBB8 leads to increased aneuploidy in mouse oocytes
Source: Cell Discov. 2023 Oct 24;9:105. doi: 10.1038/s41421-023-00599-z (PMC10598138; doi:10.1038/s41421-023-00599-z)
Supplement: Supplementary file 1 — TUBB8-Supplemental Materials [file 41421_2023_599_MOESM1_ESM.pdf]

Supplementary Materials for

Ectopic expression of human *TUBB8* leads to increased aneuploidy in mouse oocytes

Jie Dong *et al.*

* Correspondence: Lei Wang, wangleiwanglei@fudan.edu.cn;

Tianyu Wu, ty_wu@fudan.edu.cn;

Qing Sang, sangqing@fudan.edu.cn;

This PDF file includes:

Figures S1 to S5

Table S1

Supplementary Figures

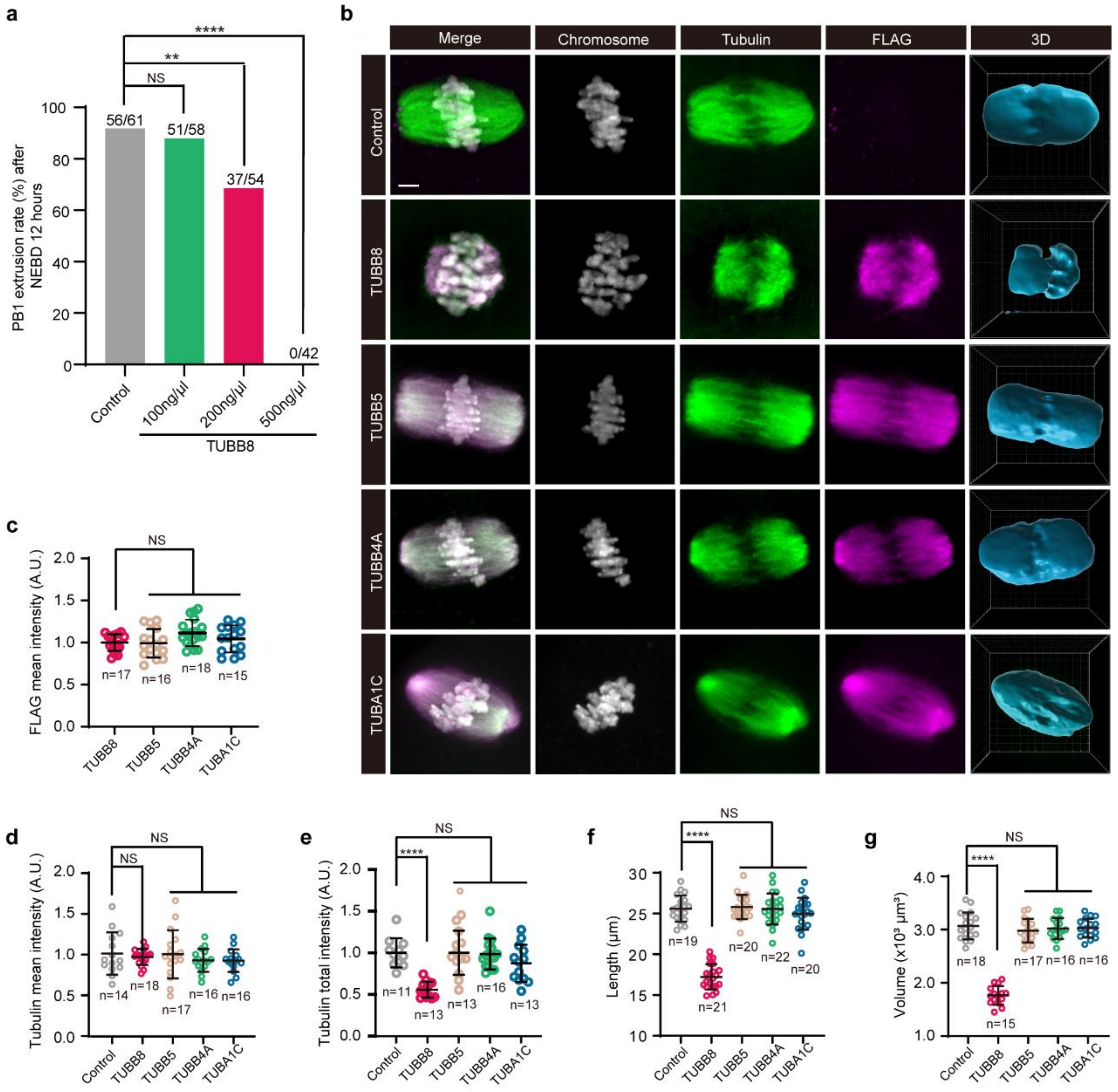

Fig. S1 Ectopic expression of *TUBB8* led to shorter spindle lengths and smaller volumes in mouse oocytes. a Statistical analysis of PB1 extrusion rates in mouse oocytes injected with varying concentrations of *TUBB8* cRNAs at 12 h after NEBD. Mouse GV oocytes were injected with 5’FLAG-*TUBB8* cRNA at varying concentrations of 100 ng/μL, 200 ng/μL and 500 ng/μL, respectively, maintained in 2.5 μM milrinone during the injection, and then washed and transferred into milrinone-free M2 medium to allow the resumption of meiosis. At 12 h after NEBD, PB1 extrusion rates in the different groups were analyzed. **b** Representative two and three-dimensional

images of spindles in MI oocytes from mouse oocytes expressing different human tubulin isotypes. Control indicates mouse GV oocytes injected with vehicle; TUBB8, TUBB5, TUBB4A and TUBA1C indicate mouse GV oocytes injected with the corresponding 5'FLAG-cRNA (200 ng/μL) of human tubulin isotypes, respectively. Mouse GV oocytes were injected with different cRNAs, maintained for 1 h in 2.5 μM milrinone, and then released into milrinone-free M2 medium to allow the resumption of meiosis. At 5 h after NEBD, oocytes were fixed and immunostained for tubulin, FLAG, and DNA (using Hoechst). Scale bar, 5 μm. **c** Statistical analysis of mean intensities of FLAG in mouse oocytes expressing different human tubulin isotypes. **d, e** Statistical analysis of mean intensities (**d**) and total intensities (**e**) of microtubules in mouse oocytes expressing different human tubulin isotypes. **f, g** Statistical analysis of spindle length (**f**), spindle volume (**g**) in mouse oocytes expressing different human tubulin isotypes. n, number of oocytes. Data in **a** were compared using Fisher's exact test. Data in **c** and **g** were tested using one-way ANOVA with multiple comparisons test. $^{**}P < 0.01$, $^{****}P < 0.0001$, NS, not significant. Data in **a**, **c**, **d**, **e**, **f** and **g** were collected from at least three independent experiments.

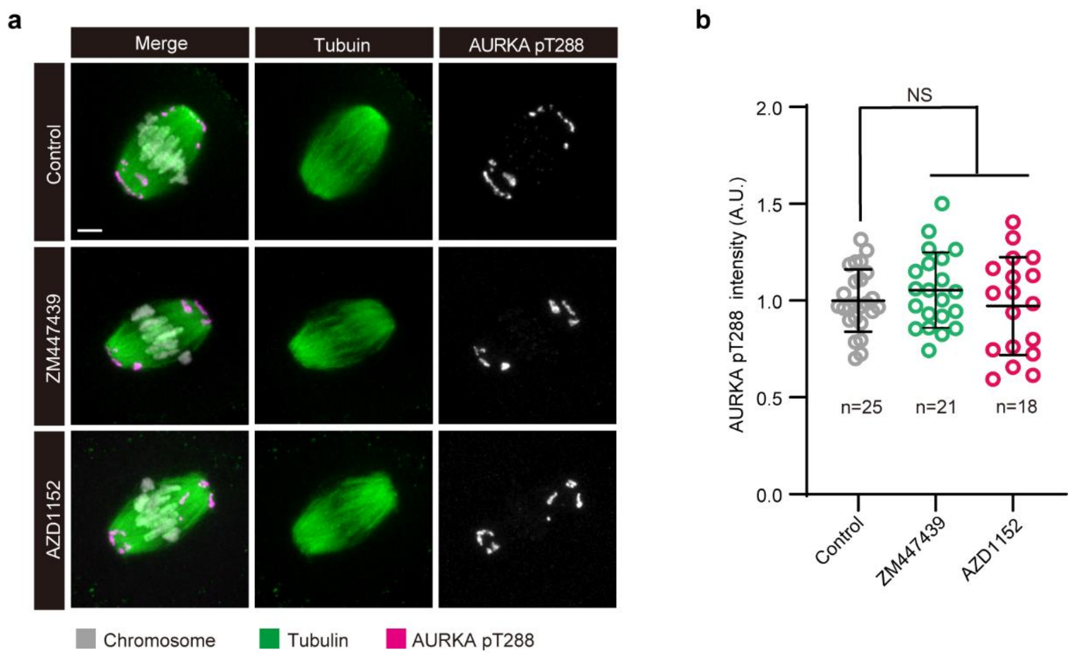

Fig. S2 ZM447439 and AZD1152 did not inhibit the activity of AURKA in the short-term treatment. a Representative images of AURKA activity as indicated by the distribution of AURKA pThr288 at late metaphase I stage. Mouse oocytes at 5 h after NEBD were treated either with ZM447439 (10 µM) or AZD1152 (500 nM) for 15 min or 30 min, and then fixed and immunostained for AURKA pThr288, tubulin, and DNA (using Hoechst). Scale bar, 5 µm. **b** Statistical analysis of AURKA pThr288 signal intensities relative to tubulin. n, number of oocytes. Differences between each group were tested using one-way ANOVA with multiple comparisons test. NS, not significant.

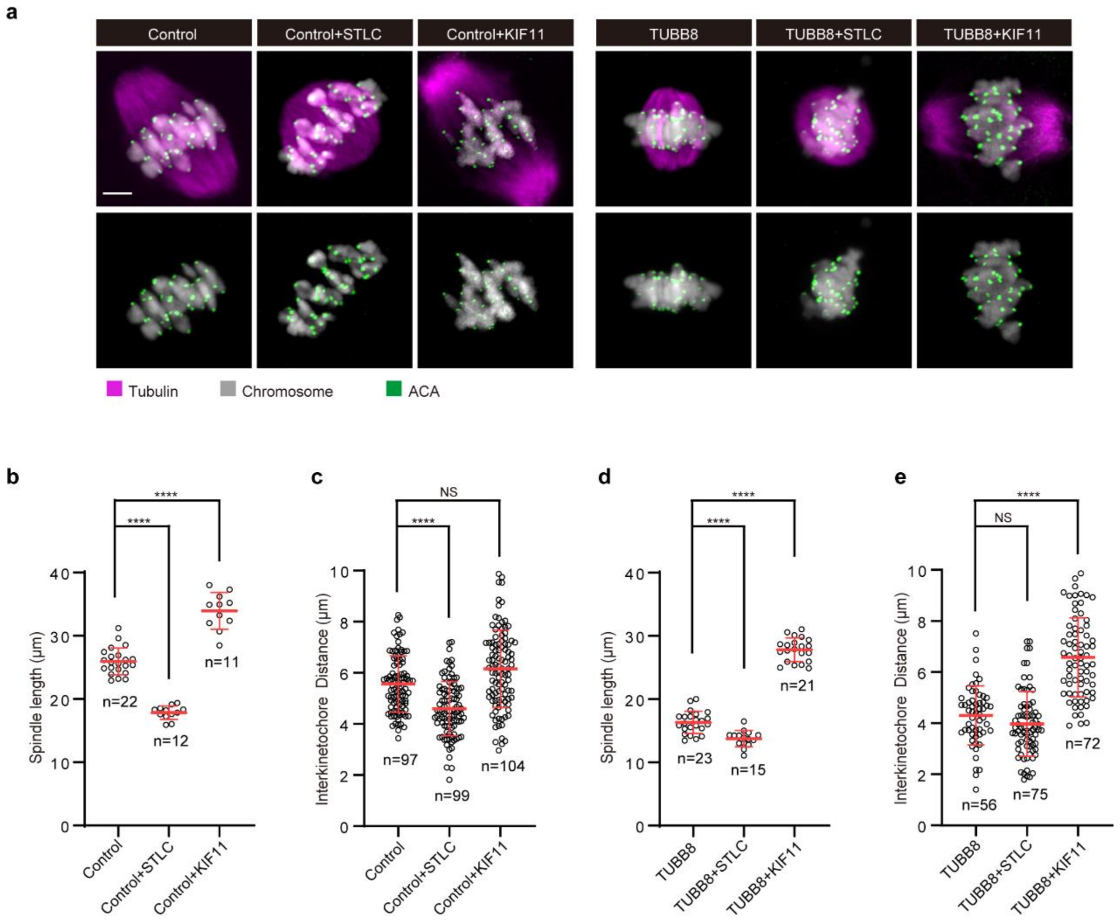

Fig. S3 Ectopic expression of *KIF11* could partly rescue the phenotypes caused by *TUBB8* ectopic expression. a Representative images of MI spindles in control oocytes (left) and oocytes expressing *TUBB8* (right) after expressing *KIF11* or being treated with STLC (0.75 μM). Mouse GV oocytes were injected with vehicle, 5'FLAG-*KIF11*-cRNA (500 ng/μL), 5'FLAG-*TUBB8*-cRNA (200 ng/μL), or a combination of 5'FLAG-*KIF11*-cRNA (1000 ng/μL) and *TUBB8*-cRNA (400 ng/μL), maintained for 2 h in 2.5 μM milrinone, and then washed and transferred into milrinone-free M2 medium to allow for the resumption of meiosis. At 5 h after NEBD, oocytes were fixed and immunostained for tubulin, ACA and DNA (using Hoechst). Scale bar, 5 μm. **b, c** Statistical analysis of the MI spindle length (**b**) and the interkinetochore distance (**c**) in controls, oocytes expressing *KIF11*, and oocytes treated with STLC. n, number of

oocytes. **d, e** Statistical analysis of the MI spindle length (**d**) and the interkinetochore distance (**e**) in mouse oocytes expressing *TUBB8*, co-expressing *TUBB8* and *KIF11*, and expressing *TUBB8* along with STLC treatment. n, number of bivalents. Data in **b**, **c**, **d** and **e** were compared using one-way ANOVA with multiple comparisons test. ****$P < 0.001$. Data in **b**, **c**, **d** and **e** were collected from at least three independent experiments.

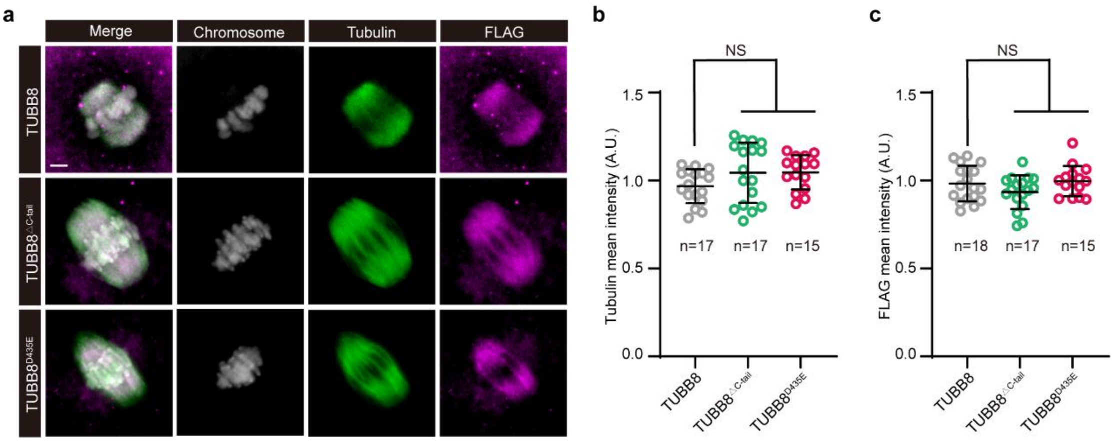

Fig. S4 *TUBB8* without the C-terminal tail and D435E variant were all incorporated into spindles equally as well as full-length *TUBB8*. a Representative images of spindles in MI oocytes from mouse oocytes expressing human full length *TUBB8* (*TUBB8*), *TUBB8* without C-terminal tail (*TUBB8*$^{\triangle\text{C-tail}}$) and D435E variant (*TUBB8*$^{\text{D435E}}$). Mouse GV oocytes were injected with 5’FLAG-*TUBB8* cRNA, 5’FLAG- *TUBB8*$^{\triangle\text{C-tail}}$ cRNA, or 5’FLAG- *TUBB8*$^{\text{D435E}}$ cRNA (200 ng/μL), maintained for 1 h in 2.5 μM milrinone, and then washed and transferred into milrinone-free M2 medium to allow the resumption of meiosis. At 5 h after NEBD, oocytes were fixed and immunostained for tubulin, FLAG, and DNA (using Hoechst). Scale bar, 5 μm. **b, c** Statistical analysis of mean intensities of microtubules (**b**) and FLAG (**c**) in mouse oocytes expressing human full length *TUBB8*, *TUBB8* without C-terminal tail and D435E variant. n, number of oocytes. Differences between each group were tested using one-way ANOVA with multiple comparisons test. NS, not significant. Data in **b** and **c** were collected from at least three independent experiments.

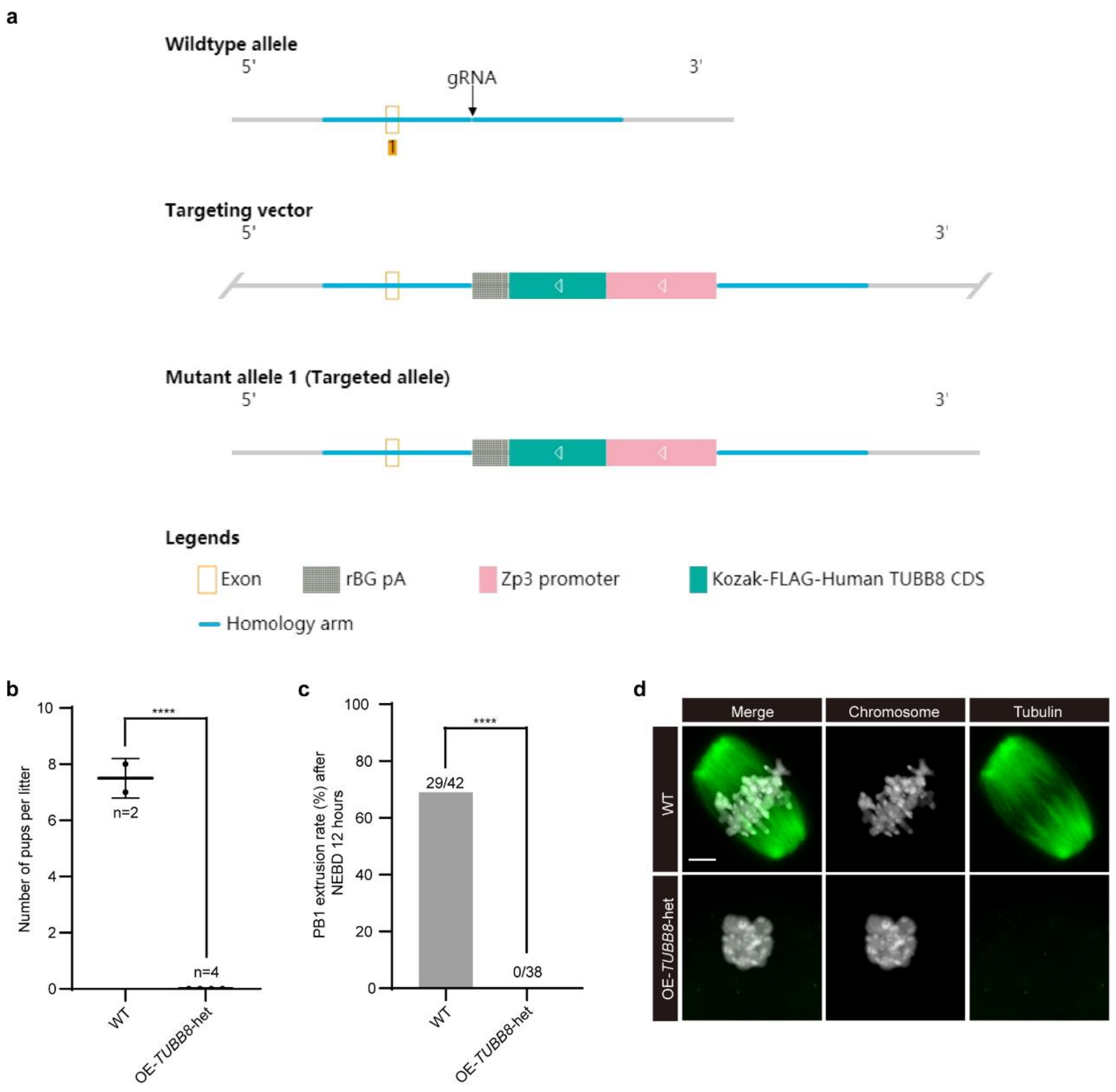

Fig. S5 Spindles can’t assemble in mouse model with oocyte-specific ectopic expression of 5’FLAG-Human-*TUBB8*. a Schematic diagram of the in vivo mice model with oocyte-specific ectopic expression of 5’FLAG-Human-*TUBB8* using a *Rosa26*-targeted *ZP3* promoter-driven KI system. **b** Reproductive ability of wildtype and heterozygous female mice expressing *TUBB8* (OE-*TUBB8*-het). Data were analyzed by Student’s t-test, n, number of mice, ****P < 0.0001. **c** Statistical analysis of PB1 extrusion rates in oocytes from wildtype and OE-*TUBB8*-het mice after NEBD 12 h. GV oocytes were isolated from the ovaries and transferred into M2 medium to allow the resumption of meiosis. At 12 h after NEBD, PB1 extrusion rates of different

groups were analyzed. Data were compared using Fisher's exact test, ****$P < 0.0001$.

d Representative images of MI spindles in wildtype and OE-*TUBB8*-het mice. GV oocytes were isolated from the ovaries and transferred into M2 medium to allow the resumption of meiosis. At 5 h after NEBD, oocytes were fixed and immunostained for tubulin and DNA (using Hoechst), Scale bar, 5 μm.

Supplementary Tables

Table S1 Primers used to amplify human genomic DNA of *TUBB8*

Mutation in cDNA	Primer name	Primer (5' to 3')
c.1305T>G<br>c.1304_1305insGGA<br>c.1311_1312insGAGGATGAGGAG	*TUBB8*-Exon4-F	AGGTGAGGAGTTACTGATGTAAAC
	TUBB8-Exon4-R	GGAGAACACTGTCCGTGCAT
